# Maternal pre-pregnancy/early-pregnancy smoking and risk of congenital heart diseases in offspring: A prospective cohort study in Central China

**DOI:** 10.7189/jogh.12.11009

**Published:** 2022-08-03

**Authors:** Tingting Wang, Lizhang Chen, Bin Ni, Xiaoqi Sheng, Peng Huang, Senmao Zhang, Jiabi Qin

**Affiliations:** 1 NHC Key Laboratory of Birth Defect for Research and Prevention, Hunan Provincial Maternal and Child Health Care Hospital, Changsha, Hunan, China; 2 Department of Epidemiology and Health Statistics, Xiangya School of Public Health, Central South University, Changsha, Hunan, China; 3 Hunan Provincial Key Laboratory of Clinical Epidemiology, Changsha, Hunan, China; 4 Department of Thoracic Cardiac Surgery, Hunan Children's Hospital, Changsha, Hunan, China

## Abstract

**Background:**

Prior studies suggested that maternal smoking before and during pregnancy could be associated with increased risks of congenital heart diseases (CHDs) in offspring. However, the results were inconsistent, and the existence of a causal relationship was not confirmed. Our study aimed to estimate the associations of maternal active and passive smoking during the pre-pregnancy/early-pregnancy period with CHDs as well as its common phenotypes in offspring.

**Methods:**

This study was based on data from a prospective cohort study conducted in Central China. A total of 49 158 eligible pregnant women between the 8th and 14th weeks of gestation were invited to join the cohort and were planned to be followed up until 3 months postpartum. The exposure of interest was maternal smoking status, including active and passive smoking status in 3 months before pregnancy as well as in early pregnancy. Self-reported maternal smoking status was ascertained via an in-person interview after recruitment. CHDs were diagnosed by pediatric cardiologists and classified according to ICD-10. Multivariable Poisson regression models were used to estimate the relative risks (RRs) with 95% confidence intervals (CIs) of all CHDs and their common phenotypes associated with maternal smoking status, adjusting for potential confounding factors identified by directed acyclic graphs.

**Results:**

CHDs were diagnosed in 564 children. After adjusting for potential confounding factors and comparing with the unexposed groups, CHDs incidence was 165% higher (adjusted RR = 2.65; 95% CI = 1.76-3.98) in offspring exposed to maternal active smoking in 3 months before pregnancy, 69% higher (adjusted-RR = 1.69; 95% CI = 1.39-2.05) in offspring exposed to maternal passive smoking in 3 months before pregnancy, 133% higher (adjusted RR = 2.33; 95% CI = 1.46-3.70) for offspring exposed to maternal active smoking in early pregnancy, and 98% higher (adjusted-RR = 1.98; 95% CI = 1.56-2.51) for offspring exposed to maternal passive smoking in early pregnancy. More specifically, the offspring exposed to maternal active smoking in early pregnancy had the highest risk of Tetralogy of Fallot (adjusted RR = 9.84; 95% CI = 2.49-38.84). These findings were recapitulated in analyses that further adjusted for other behaviour variables apart from the characteristic being assessed and were also confirmed by sensitivity analyses.

**Conclusions:**

Our findings add to the existing body of evidence that implicates maternal pre-pregnancy/early-pregnancy smoking as a significant risk factor for CHDs and their select phenotypes.

Reports project that approximately 1 in 5 women of reproductive age will be tobacco users by the end of 2025 [1]. Although strategies to prevent cigarette smoking have been implemented globally, about 3.0% of pregnant women in China smoke during pregnancy and up to 25% of women in the United States are reported to have various degrees of tobacco consumption [2]. Cigarette smoke during pregnancy is a major global public health issue, not only because it influences women’s own health, but because it is also related to adverse offspring outcomes, such as congenital malformations and stillbirth [3,4]. Maternal passive smoking (also known as second-hand smoke exposure) has also been reported as relevant for poorer health outcomes in the perinatal period [5]. These harmful effects are mediated by the toxic effects of carbon monoxide, nicotine, and other cigarette components which can pass the placental barrier [6].

As the most common group of congenital malformations among newborns, congenital heart diseases (CHDs) have been the focus of research efforts on maternal and child health due to their contribution to infant mortality and morbidity and their impact on the long-term health of affected individuals [7-9]. However, the cause of CHD is still unclear and primary prevention strategies are unavailable. Numerous researchers have attempted to determine the relationship between prenatal tobacco exposure and CHDs over the last few years, with mixed results. The causal relationship between the observed increased risks of CHDs in offspring and prenatal exposure to tobacco exposure is also ambiguous. A recent meta-analysis on the association of prenatal tobacco exposure to CHDs found that maternal active and passive smoking were significantly associated with CHDs risks, with relative risks (RRs) of 1.09 and 1.57, respectively [10]. However, the results of this study must be interpreted with caution. Data were primarily sourced from case-control studies (86.4%), and the status of prenatal tobacco exposure was predominantly based upon retrospective data, which were subject to recall bias. Additionally, 68% of the included studies did not adjust for any confounding factors. Accordingly, the study could not determine whether the observed association between maternal cigarette smoking and CHDs reflected the magnitude of a causal effect or was biased by confounding factors. Considering that the critical development period of CHDs is from the 3rd to the 8th week of gestation, studies on risk factors for CHDs should focus on the critical period, that is, from 3 months before pregnancy to the end of the first trimester [11]. However, 70.4% of the studies within the meta-analysis did not explicitly define the time of tobacco exposure, which may further confound the observed associations between maternal cigarette smoking and risks of CHDs in offspring [10].

Given the relatively high prevalence of prenatal tobacco exposure and the potential severity of CHDs, it is necessary to clarify the association between maternal cigarette smoking (including active and passive smoking) and risk of CHDs. In this prospective cohort study, data derived from 44 048 eligible pregnant women and their offspring were analysed. The objective was to estimate the relative risks of CHDs in relation to maternal active and passive smoking in 3 months before pregnancy and in early pregnancy. Considering the potential etiological heterogeneity between different phenotypes, the relative risks of specific CHD phenotypes were also estimated.

## METHODS

### Study population and information collection

**Figure 1:**
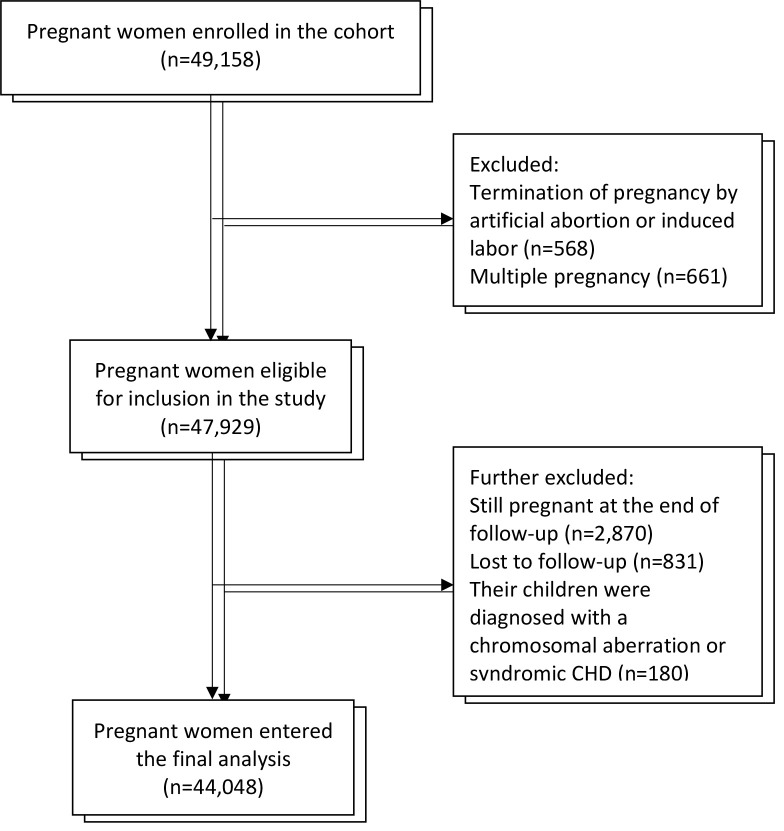
Flowchart illustrating the process of participant recruitment. CHDs – congenital heart diseases.

Pregnant women of 18 years or older, receiving their first antenatal care between the 8th and 14th weeks of gestation, and intending to deliver at the study hospital were approached and invited to participate in the cohort between March 13, 2013, to December 31, 2019. The gestational week was estimated using the self-reported data on last menstrual period or calculated by ultrasound if menstrual irregularities were present [12]. After written informed consent was obtained, a total of 49 158 eligible pregnant women were recruited. After excluding participants who had multiple pregnancies (n = 661), terminated a pregnanc by artificial abortion or induced labour (n = 568), were still pregnant at the end of follow-up or lost to follow-up (n = 3701), and those whose offspring were diagnosed with chromosomal aberrations or syndromic CHDs (n = 180), a total of 44 048 pregnant women were retained (**Figure 1**).

Face-to-face interviews were administered by trained investigators to collect information on exposures and background factors from all participants. The participants were subsequently followed up until 3 months after birth to collect detailed information on infant illness, particularly on birth defects. Information on disease diagnosis was confirmed by medical records.

### Exposures and background factors

The exposure of interest was maternal smoking status, including active smoking in 3 months before pregnancy, passive smoking in 3 months before pregnancy, active smoking in early pregnancy, and passive smoking in early pregnancy. Active smoking was defined by a consumption of at least one cigarette during the target period, while passive smoking was defined as exposure to another person’s tobacco smoke for at least 15 minutes daily for more than one day per week during the target period.

Information on background factors included socio-demographic characteristics, obstetric, clinical, and genetic characteristics, disease and health conditions, behaviour characteristics, and exposure to environmentally harmful substances. Details are shown in **Table 1**. “Minority” refers to the other 55 ethnicities in Hunan Province, except in Han. Body mass index was calculated as the self-reported body weight in kilograms divided by the self-reported body height in meters squared. Alcohol consumption was defined as consuming at least one standard alcoholic drink during the target period.

**Table 1:** Data collection from the participants

Category	Variable
Socio-demographic characteristics	Age (<25, 25-29.9, 30-34.9, and ≥35 y), ethnicity (Han and minority), and educational level (junior high school or below, senior middle school, college, and master or above)
Obstetric, clinical, and genetic characteristics	Mode of conception (spontaneous conception and assisted conception), consanguineous marriage (ie, yes or no), parity (primiparous and multiparous), history of adverse pregnancy outcomes (ie, yes or no), and family history of congenital malformations (ie, yes or no)
Disease and health conditions	Pre-pregnancy body mass index (<18.5 kg/m^2^, 18.5-23.9 kg/m^2^, 24-27.9 kg/m^2^, and ≥28 kg/m^2^), pre-pregnancy diabetes mellitus (ie, yes or no), and personal history of congenital malformations (ie, yes or no)
Behaviour characteristics	Taking folic acid in 3 mo before pregnancy or in early pregnancy (ie, yes or no), alcohol consumption in 3 mo before pregnancy (ie, yes or no), and alcohol consumption in early pregnancy (ie, yes or no)
Exposure of environmentally harmful substances	Exposure to environmental pollution around the dwelling place in three months before pregnancy or in early pregnancy (ie, yes or no), and exposure to radioactive hazardous while at work in three months before pregnancy or in early pregnancy (ie, yes or no).

### Outcomes

The outcome of interest was CHDs diagnosed by paediatric cardiologists through physical examination, cardiac auscultation, pulse oximetry, electrocardiogram, and echocardiography. The classifications of CHDs were based on the International Classification of Diseases-10 (ICD-10) codes (Q20-Q28). CHD phenotypes, including transposition of the great arteries (TGA, Q20.3), atrial septal defect (ASD, Q21.0), ventricular septal defect (VSD, Q21.1), atrioventricular septal defect (AVSD, Q21.2), patent ductus arteriosus (PDA, Q25.0), and pulmonary stenosis (PS, Q25.6), were also assessed in this study.

### Statistical analysis

This study was reported following the STROBE statement [13]. To avoid data entry errors such as out-of-range values and mismatches, all data were entered using a double-check strategy to assure accuracy of the data in EpiData 3.1 (EpiData Association, Odense, Denmark). A directed acyclic graph (DAG) was constructed by using the online DAGitty software (http://www.dagitty.net/) to select covariates for inclusion in the statistical models [14]. The relationships between each of the variables were assigned based on knowledge of the publications regarding these associations. Concerning assumptions described in the DAG theory, a minimal sufficient set of adjustment variables was identified for estimating the effect of maternal smoking on offspring risk of CHDs. The DAGs and variables in the minimal sufficient adjustment sets are shown in Figures S1-S4 in the **Online Supplementary Document**.

Descriptive statistics were employed to assess participant characteristics and χ^2^ tests were applied to compare categorical variables. Multivariable Poisson regression models were used to estimate RRs and 95% confidence intervals (CIs) for CHDs in offspring who were exposed to maternal smoking during the peri-pregnancy period [15]. These variables in the minimal sufficient adjustment set identified with DAGs were included as covariates. The behavioural characteristics that have not been identified in DAGs were further adjusted. Specifically, the variables “passive smoking in 3 months before pregnancy” and “alcohol drinking in 3 months before pregnancy” were added as covariates for analysis of maternal active smoking in 3 months before pregnancy as the exposure variable, the variables “active smoking in 3 months before pregnancy” and “alcohol drinking in 3 months before pregnancy” were added as covariates for analysis of maternal passive smoking in 3 months before pregnancy as the exposure variable, the variables “passive smoking in early pregnancy” and “alcohol drinking in early pregnancy” were added as covariates for analysis of maternal active smoking in early pregnancy as the exposure variable, while the variables “active smoking in early pregnancy” and “alcohol drinking in early pregnancy” were added as covariates for analysis of maternal passive smoking in early pregnancy as the exposure variable. Although the variables “active smoking in early pregnancy”, “passive smoking in early pregnancy”, and “alcohol drinking in early pregnancy” were identified as mediator variables of the effect of maternal active smoking in 3 months as well as maternal passive smoking before pregnancy on offspring risk of CHDs based on the DAG, we did not adjust the mediators in the regression model since the primary aim of this study was to assess the overall effect of maternal smoking status.

Sensitivity analysis was performed by re-analysing the association of maternal smoking status with offspring risk of CHDs after excluding pregnant women whose offspring had any congenital disease other than CHDs. All the statistical analyses were conducted by using SPSS 20.0 (IBM SPSS Inc., Chicago, USA) and a *P* ≤ 0.05 was considered statistically significant.

### Ethics approval

This study was based on data from a prospective cohort study of pregnant women and their offspring conducted at the Hunan Provincial Maternal and Child Health Care Hospital. This study received ethics approval from the Ethics Committee of Xiangya School of Public Health of Central South University. Each participant was informed of the study protocol and the voluntary nature of participation and provided written informed consent prior to completing an interviewer-administrated questionnaires and providing biological samples (eg, blood sample).

## RESULTS

### Sample characteristics

Among the eligible pregnant women with singleton pregnancies (n = 44 048), 716 (1.6%) reported actively smoking in 3 months before pregnancy, 6524 (14.8%) reported passively smoking in 3 months before pregnancy, 604 (1.4%) reported actively smoking in early pregnancy, while 3148 (7.1%) reported passively smoking in early pregnancy. At the end of follow-up, 564 infants had been diagnosed with any form of non-syndromic CHD. The most common diagnoses in these infants were ASD (n = 170; 12.8 per 1000, 95% CI = 11.8-13.9 per 1000), VSD (n = 246; 3.9 per 1000, 95% CI = 3.3-4.4 per 1000), AVSD (n = 52; 5.6 per 1000, 95% CI = 4.9-6.3 per 1000), PDA (n = 84; 1.9 per 1000, 95% CI = 1.5-2.3 per 1000), TOF (n = 36; 0.8 per 1000, 95% CI = 0.6-1.1 per 1000), PS (n = 44; 1.0 per 1000, 95% CI = 0.1-1.3 per 1000), and TGA (n = 28; 0.6 per 1000, 95% CI = 0.4-0.9 per 1000).

The distribution of baseline characteristics is summarised in **Table 2**. The participants were concentrated 25 to 29.9 years (44.8%) and 30 to 34.9 years (32.2%) age groups and their educational levels were mostly senior middle school (52.6%) and college (24.6%). They were mostly of Han nationality (94.9%), natural conception (77.4%), and multipara (55.6%). More than 40% of the pregnant women reported a history of adverse pregnancy outcomes. Relatively few reported a personal history of pre-pregnancy diabetes mellitus (0.8%) or congenital malformations (1.1%), ever consuming alcohol weekly in 3 months before pregnancy (2.0%) or in early pregnancy (1.3%), or being exposed to environmental pollution around the dwelling place (2.1%) or radioactive hazards at work (3.3%) in 3 months before pregnancy or in early pregnancy. The distribution of baseline characteristics by tobacco smoking status was also summarised in **Table 2**.

**Table 2:** Baseline characteristics of the study participants

Baseline characteristics	Total (n, %)	Active smoking in 3 mo before pregnancy			Passive smoking in 3 mo before pregnancy			Active smoking in early pregnancy			Passive smoking in early pregnancy		
		**No (n, %)**	**Yes (n, %)**	***P*-value**	**No (n, %)**	**Yes (n, %)**	***P*-value**	**No (n, %)**	**Yes (n, %)**	***P*-value**	**No (n, %)**	**Yes (n, %)**	***P*-value**
**Sociodemographic characteristics**													
Age (years)				<0.001			<0.001			0.909			<0.001
<25	4980 (11.3%)	4954 (11.4%)	26 (3.6%)		4386 (11.7%)	594 (9.1%)		4914 (11.3%)	66 (10.9%)		4720 (11.5%)	260 (8.3%)	
25-29.9	19 724 (44.8%)	19 510 (45.0%)	214 (29.9%)		16 796 (44.8%)	2928 (44.9%)		19 460 (44.8%)	264 (43.7%)		18 280 (44.7%)	1444 (45.9%)	
30-34.9	14 192 (32.2%)	13 822 (31.9%)	370 (51.7%)		12 004 (32.0%)	2188 (33.5%)		13 990 (32.2%)	202 (33.4%)		13 150 (32.2%)	1042 (33.1%)	
≥35	5152 (11.7%)	5046 (11.6%)	106 (14.8%)		4338 (11.6%)	814 (12.5%)		5080 (11.7%)	72 (11.9%)		4750 (11.6%)	402 (12.8%)	
Ethnicity				0.001			0.184			0.311			<0.001
Han	41 818 (94.9%)	41 158 (95.0%)	660 (92.2%)		35 646 (95.0%)	6172 (94.6%)		41 250 (94.9%)	568 (94.0%)		38 910 (95.1%)	2908 (92.4%)	
Minority	2230 (5.1%)	2174 (5.0%)	56 (7.8%)		1878 (5.0%)	352 (5.4%)		2194 (5.1%)	36 (6.0%)		1990 (4.9%)	240 (7.6%)	
Educational level				<0.001			0.731			0.152			0.046
Junior high school or below	6900 (15.4%)	6678 (15.4%)	222 (31.0%)		5896 (15.7%)	1004 (15.4%)		6822 (15.7%)	78 (12.9%)		6378 (15.6%)	522 (16.6%)	
Senior middle school	23 164 (52.6%)	22 760 (52.5%)	404 (56.4%)		19 752 (52.6%)	3412 (52.3%)		22 850 (52.6%)	314 (52.0%)		21 580 (52.8%)	1584 (50.3%)	
College	10 820 (24.6%)	10 730 (24.8%)	90 (12.6%)		9192 (24.5%)	1628 (25.0%)		10 654 (24.5%)	166 (27.5%)		10 026 (24.5%)	794 (25.2%)	
Master or above	3164 (7.2%)	3164 (7.3%)	0 (0.0%)		2684 (7.2%)	480 (7.4%)		3118 (7.2%)	46 (7.6%)		2916 (7.1%)	248 (7.9%)	
**Obstetric, clinical, and genetic characteristics**													
Mode of conception				<0.001			<0.001			0.181			<0.001
Spontaneous conception	34 104 (77.4%)	33 762 (77.9%)	342 (47.8%)		29 166 (77.7%)	4938 (75.7%)		33 650 (77.5%)	454 (75.2%)		31 752 (77.6%)	2352 (74.7%)	
Assisted conception	9944 (22.6%)	9570 (22.1%)	374 (52.2%)		8358 (22.3%)	1586 (24.3%)		9794 (22.5%)	150 (24.8%)		9148 (22.4%)	796 (25.3%)	
Parity				0.447			0.679			0.853			0.603
Primipara	19 562 (44.4%)	19 234 (44.4%)	328 (45.8%)		16 680 (44.5%)	2882 (44.2%)		19 296 (44.4%)	266 (44.0%)		18 150 (44.4%)	1412 (44.9%)	
Multipara	24 486 (55.6%)	24 098 (55.6%)	388 (54.2%)		20 844 (55.5%)	3642 (55.8%)		24 148 (55.6%)	388 (56.0%)		22 750 (55.6%)	1736 (55.1%)	
Consanguineous marriage (yes)	184 (0.4%)	178 (0.4%)	6 (0.8%)	0.079	156 (0.4%)	28 (0.4%)	0.876	172 (0.4%)	12 (2.0%)	<0.001	164 (0.4%)	20 (0.6%)	0.049
History of adverse pregnancy outcomes (yes)	17 758 (40.3%)	17 434 (40.2%)	324 (45.3%)	0.007	15 146 (40.4%)	2612 (40.0%)	0.620	17 522 (40.3%)	236 (39.1%)	0.531	16 512 (40.4%)	1246 (39.6%)	0.383
Family history of congenital malformations (yes)	62 (0.1%)	54 (0.1%)	8 (1.1%)	<0.001	54 (0.1%)	8 (0.1%)	0.672	62 (0.1%)	0 (0.0%)	0.353	52 (0.1%)	10 (0.3%)	0.006
Disease and health conditions													
Pre-pregnancy BMI (kg/m^2^)				<0.001			0.672			0.738			0.005
<18.5	7532 (17.1%)	7292 (16.8%)	240 (33.5%)		6382 (17.0%)	1150 (17.6%)		7434 (17.1%)	98 (16.2%)		6982 (17.1%)	550 (17.5%)	
18.5-23.9	29 324 (66.6%)	28 952 (66.8%)	372 (52.0%)		25 008 (66.6%)	4316 (66.2%)		28 916 (66.6%)	408 (67.5%)		27 300 (66.7%)	2024 (64.3%)	
24-27.9	6062 (13.8%)	5976 (13.8%)	86 (12.0%)		5168 (13.8%)	894 (13.7%)		5976 (13.8%)	86 (14.2%)		5566 (13.6%)	496 (15.8%)	
≥28	1130 (2.6%)	1112 (2.6%)	18 (2.5%)		966 (2.6%)	164 (2.5%)		1118 (2.6%)	12 (2.0%)		1052 (2.6%)	78 (2.5%)	
Pre-pregnancy diabetes mellitus (yes)	322 (0.8%)	322 (0.7%)	10 (1.4%)	0.045	248 (0.7%)	84 (1.3%)	<0.001	328 (0.8%)	4 (0.7%)	0.794	314 (0.8%)	18 (0.6%)	0.221
Personal history of congenital malformations (yes)	466 (1.1%)	466 (1.1%)	0 (0.0%)	0.005	390 (1.0%)	76 (1.2%)	0.360	464 (1.1%)	2 (0.3%)	0.079	432 (1.1%)	34 (1.1%)	0.900
**Behaviour characteristics**													
Taking folic acid in 3 mo before pregnancy or in early pregnancy (yes)	42 072 (95.5%)	1942 (4.5%)	34 (4.7%)	0.732	1670 (4.5%)	306 (4.7%)	0.388	1942 (4.5%)	34 (5.6%)	0.172	1784 (4.4%)	192 (6.1%)	<0.001
Alcohol drinking in 3 mo before pregnancy (yes)	894 (2.0%)	772 (1.8%)	122 (17.0)	<0.001	798 (2.1%)	96 (1.5%)	0.001	886 (2.0%)	8 (1.3%)	0.216	836 (2.0%)	58 (1.8%)	0.440
Alcohol drinking in early pregnancy (yes)	564 (1.3%)	526 (1.2%)	38 (5.3%)	<0.001	498 (1.3%)	66 (1.0%)	0.036	554 (1.3%)	10 (1.7%)	0.409	514 (1.3%)	50 (1.6%)	0.111
**Exposure of environmentally harmful substances**													
Exposure to environmental pollution around the dwelling place in three months before pregnancy or in early pregnancy (yes)	920 (2.1%)	902 (2.1%)	18 (2.5%)	0.422	794 (2.1%)	126 (1.9%)	0.336	892 (2.1%)	28 (4.6%)	<0.001	858 (2.1%)	62 (2.0%)	0.628
Exposure to radioactive hazardous while at work in three months before pregnancy or in early pregnancy (yes)	1432 (3.3%)	1416 (3.3%)	16 (2.2%)	0.122	1220 (3.3%)	212 (3.2%)	0.994	1414 (3.3%)	18 (3.0%)	0.705	1328 (3.2%)	104 (3.3%)	0.863

BMI – body mass index, mo – months

### Maternal smoking status in 3 months before pregnancy and the risk of CHDs in offspring

**Figure 2:**
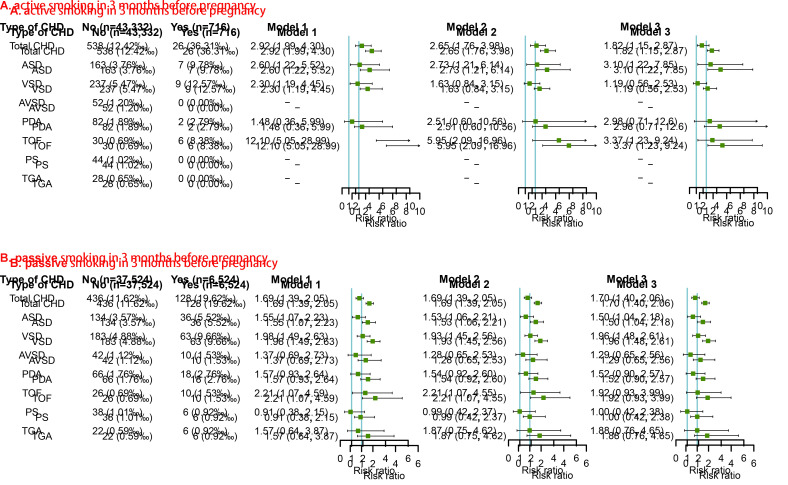
Maternal smoking status in 3 months before pregnancy and risk of CHDs and its phenotypes in offspring. A) Covariates including age, ethnicity, educational level, parity, history of adverse pregnancy outcomes, and pre-pregnancy BMI were adjusted. B) Covariates including age, ethnicity, educational level, parity, and history of adverse pregnancy outcomes were adjusted. BMI – body mass index, CHDs – congenital heart defects, RR – risk ratio.

The associations between maternal smoking status in 3 months before pregnancy and risk of CHDs in offspring are presented in **Figure 2**. After adjusting for potential confounders identified by DAGs (Model 2 in **Figure 2****,** panel A), maternal active smoking was independently associated with increased risks of total CHD (RR = 2.65, 95% CI = 1.76-3.98), ASD (RR = 2.73, 95% CI = 1.21-6.14), and TOF (RR = 5.95, 95% CI = 2.09-16.96) in offspring. Further adjustment for other behavioural characteristics including passive smoking in 3 months before pregnancy and alcohol consumption in 3 months before pregnancy did not materially alter the risks estimates for total CHD, ASD, and TOF in offspring of pregnant women who had ever actively smoked (Model 3 in **Figure 2** panel A).

After adjusting for potential confounders identified by DAGs (Model 2 in **Figure 2** panel B), the risk estimates of total CHD (RR = 1.69, 95% CI = 1.39,2.05), ASD (RR = 1.53, 95% CI = 1.06-2.21), VSD (RR = 1.93, 95% CI = 1.45-2.56), and TOF (RR = 2.21, 95% CI = 1.07-4.55) significantly increased in offspring of pregnant women who had ever passively smoked in 3 months before pregnancy. Further adjustment for the behavioural characteristic - active smoking in 3 months before pregnancy and alcohol consumption in 3 months before pregnancy - also showed increased risks of total CHD, ASD, and VSD (Model 3 in **Figure 2** panel B).

### Maternal smoking status in early pregnancy and the risk of CHDs in offspring

**Figure 3:**
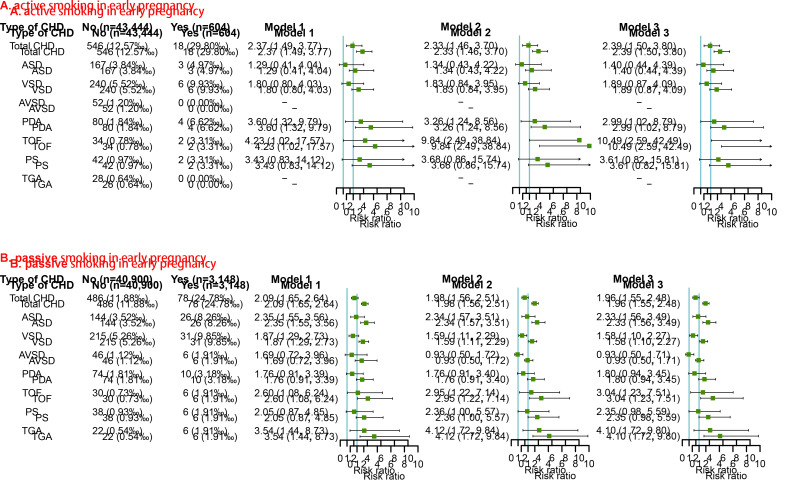
Maternal smoking status in early pregnancy and risk of CHDs and its phenotypes in offspring. A) Covariates including age, ethnicity, educational level, model of conception, parity, history of adverse pregnancy outcomes, pre-pregnancy BMI, active smoking in 3 months before pregnancy, passive smoking in 3 months before pregnancy, and alcohol consumption in 3 months before pregnancy were adjusted. B) Covariates including age, ethnicity, educational level, model of conception, parity, history of adverse pregnancy outcomes, pre-pregnancy BMI, active smoking in 3 months before pregnancy, and passive smoking in 3 months before pregnancy were adjusted. BMI – body mass index, CHDs – congenital heart defects, RR – risk ratio.

The associations of maternal smoking status in early pregnancy and risk of CHDs in offspring are presented in **Figure 3**. After adjusting for potential confounders identified by DAGs (Model 2 in **Figure 3** panel A), maternal active smoking was independently associated with increased risks of total CHD (RR = 2.33, 95% CI = 1.46-3.70), PDA (RR = 3.26, 95% CI = 1.24-8.56), and TOF (RR = 9.84, 95% CI = 2.49-38.84) in offspring. After further adjustment for other behavioural characteristics (including passive smoking in early pregnancy and alcohol consumption in early pregnancy), significantly increased risks of total CHD, PDA, and TOF were still observed in offspring of pregnant women who had ever actively smoked. (Model 3 in **Figure 3** panel A).

After adjusting for potential confounders identified by DAGs (Model 2 in **Figure 3** panel B), the risk estimates of total CHD (RR = 1.98, 95% CI = 1.56-2.51), ASD (RR = 2.34, 95% CI = 1.57-3.51), VSD (RR = 1.59, 95% CI = 1.11-2.29), TOF (RR = 2.95, 95% CI = 1.22-7.14), and TGA (RR = 4.12, 95% CI = 1.72-9.84) significantly increased in offspring of pregnant women who had ever passively smoked in early pregnancy. Further adjustment for behavioural characteristic (active smoking in early pregnancy and alcohol consumption in early pregnancy) also showed increased risks of total CHD, ASD, VSD, TOF, and TGA (Model 3 in **Figure 3** panel B).

### Sensitivity analysis

Sensitivity analysis confirmed previous results. Exclusion of pregnant women whose offspring had any congenital disease other than CHD (n = 1716) did not materially change the presented results. Data are shown in Figure S5 and Figure S6 in the **Online Supplementary Document**.

## DISCUSSION

### Principal findings

In this cohort study, after adjusting for the potential confounders, significantly higher risks of CHDs were detected in offspring exposed to maternal cigarette smoking in 3 months before pregnancy, with an increased risk of 165% for active smoking and 69% for passive smoking. Maternal cigarette smoking in early pregnancy was also independently associated with risk of CHDs in offspring, with an increased risk of 133% for active smoking and 98% for passive smoking. These findings were recapitulated in analyses that were further adjusted for the other behaviour variables, apart from the characteristic being assessed, and were also confirmed by the sensitivity analyses. For specific phenotypes, maternal active and passive smoking in each period were significantly associated with mildly-to-moderately increased risks of two or more CHD phenotypes in offspring, except for a robustly increased risk for TOF in offspring exposed to maternal active smoking in early pregnancy. Since different phenotypes of CHDs may have differential aetiologies, it is conceivable that the risk of developing each specific CHDs is inconsistent. To the best of our knowledge, this is the first prospective cohort study conducted in early pregnancy to clarify the relationship between maternal prenatal tobacco smoking and the risk of offspring CHDs in China. This study, which better qualifies and quantifies these risks, highlights the need for continued efforts to promote smoking cessation particularly during the peri-pregnancy period.

### Study strengths

The present study has several strengths, including the use of prospective cohort design to minimize recall bias and ensure the reliability of the study. Taking the pre-pregnancy and early pregnancy periods as the focus periods of our study also strengthened the reliability of the findings. Case confirmation of CHD by a panel of paediatric cardiologists involved intensive and direct clinical review, which minimized the possibility and extent of misclassification. The convenient and effective communication methods established between researchers and participants (eg, telephone call, short message, WeChat, etc.) contributed to reducing the rate of loss to follow-up, which was only 1.7% in the present study. Additionally, a rich set of variables such as age, ethnicity, educational level, parity, history of adverse pregnancy outcomes, and pre-pregnancy BMI were evaluated and included as candidate covariates in the statistical analyses, which reduced the likelihood of confounding by such factors. Different from previous studies, our study used DAGs instead of traditional methods to identify “potential confounders”; this method could minimize bias that were recently shown to be present when using the traditional methods to select confounders [16]. Thus, the increased risks of CHDs observed in our study is more likely to be an unbiased estimate of effect for maternal tobacco exposure on offspring CHDs.

### Data limitations

The study has several limitations. First, participants were recruited from a single tertiary care centre. The results of our may not reflect those of the other institutions across the country. Therefore, it is advisable to exercise caution when extrapolating these results.

Second, concerns about missing diagnosis of CHDs could be raised since the follow-up was current to 3 months postpartum. Potentially missed cases may be diluted among our large participants.

Third, maternal cigarette smoking prior to and in early pregnancy was self-reported, which may be affected by information bias. A previous study showed that there was a high correlation (r = 0.70) between the number of cigarettes reported by women themselves and objectively measured biomarker (ie, cotinine) at any given point in time, suggesting that self-reported smoking status before and during pregnancy could reflect foetal exposure in epidemiological studies [17]. Even so, we cannot confirm the validity of self-reported smoking status given that women may have been more likely to misreport their smoking behaviours, as knowledge of dangers related to smoking during pregnancy has become more widespread in recent years. However, given that information about maternal smoking was determined before the birth of a child, any misclassification is expected to be non-differential.

Fourth, for maternal pre-pregnancy smoking, only data on smoking status in 3 months before pregnancy were collected. Thus, we could not distinguish between never smokers and those who did not smoke during the last 3 months before pregnancy. Moreover, due to a lack of available data, we were unable to assess the effect of changes in the frequency and/or quantity of cigarette smoking before and during pregnancy on CHDs risk. We were also unable to determine whether there was a dose-response relationship between maternal smoking and CHDs risks. Further well-designed studies solving current limitations and knowledge gaps are warranted.

Fifth, in common with other observational studies, although severalpotential confounding factors were adjusted in the analyses, the possibility of residual confounding arising from unknown or unmeasured covariates could not be excluded.

Sixth, some of our findings might be due to chance; this is a common consideration in epidemiological studies, especially in studies involving multiple comparisons.

Another limitation was the relatively small sample size of certain exposure and cardiac defect groups, which contributed to a reduction in the precision of risk estimates and therefore the power to detect significant effects when these were present. The risk of specific CHD phenotypes should be further investigated in larger studies.

### Interpretation

Consistent with our findings, the previous meta-analysis of nearly 140 000 CHD cases reported positive associations of maternal cigarette smoking with risks of CHDs in offspring, with an increased risk of 25% for active smoking and 124% for passive smoking, respectively [10]. However, there was considerable heterogeneity in their pooled results (*I^2^* = 89%, 92%), which might result in reduced credibility of the results. Adjustment for confounding factors in the meta-analysis was poor, with 94 of the 125 included studies not adjusting for any confounders or not providing information on confounders adjustment. It’s possible that significant unmeasured confounders have contributed to the observed associations. Additionally, more than 70% of the included studies did not clearly define the time of maternal smoking [10]. Given that the critical development period of CHDs is the 3rd to 8th weeks of gestation, studies on risk factors for CHDs should focus on the critical period, that is, from 3 months before pregnancy to the end of the first trimester. Therefore, the risk estimate reported in the meta-analysis, which treated maternal smoking during the period from 6 months prior to pregnancy to the end of the third trimester as a group, may be uninformative and potentially misleading. With a more rigorous adjustment for confounding and a clearer temporal definition of maternal smoking, our study provided more robust evidence that maternal active and passive smoking in 3 months before pregnancy as well as in early pregnancy are associated with increased risks of CHDs in offspring, and these associations are unlikely to be explained by residual confounders and are potential causal.

In our study, surprisingly higher risks of TOF were found in offspring exposed to maternal active smoking in 3 months before pregnancy (RR = 5.95) and in early pregnancy (RR = 9.84). This was somewhat surprising when other phenotypes only had mild to moderate risks or even had no risks related to maternal smoking. The results should be interpreted cautiously given the small number of CHD cases in analyses assessing risks of specific CHD phenotypes. Further studies are warranted to clarify the effect of maternal smoking before and in early pregnancy on specific CHD phenotypes, which could provide deeper insights into the potential teratogenic effect of maternal prenatal tobacco exposure.

Our findings of significantly increased risks of CHDs in offspring exposed to maternal active and passive smoking before and in early pregnancy support the importance of investing in the prevention of cigarette smoking in women of childbearing age before or at the beginning of pregnancy. Preventing youth (future mothers) from smoking so that the level of smoking during pregnancy stays low would be especially important in China, where smoking during pregnancy is remarkedly lower than in USA or Europe [2]. Current guidelines focus solely on quitting smoking rather than reducing smoking [18-21], which may discourage women who feel it difficult to quit smoking cigarettes. These women should be given enough information not only on risks of continued smoking, but also on benefits of reducing the number of cigarettes [22]. Further studies should investigate whether reducing the number of cigarettes in early pregnancy is beneficial for CHDs as well as other adverse birth and offspring outcomes.

## CONCLUSIONS

Our study provided evidence to support an intrauterine effect of maternal active and passive smoking on CHDs, both in 3 months before pregnancy and in early pregnancy. Although all clinical guidelines encourage everyone not to smoke, and if women do smoke, to quit before pregnancy, there is still a considerable proportion of women smoking before or during pregnancy. More effective strategies are needed to support women of childbearing age not to start smoking and to encourage women who are smoking before pregnancy to quit or reduce smoking. Detailed insight into the specific mechanisms that link maternal smoking to the increased risk of CHD in offspring can determine targets for the preventive intervention.

## Additional material


Online Supplementary Document
