# Supplementary material for: Maternal pre-pregnancy/early-pregnancy smoking and risk of congenital heart diseases in offspring: A prospective cohort study in Central China
Source: J Glob Health. 2022 Aug 3;12:11009. doi: 10.7189/jogh.12.11009 (PMC9344981; doi:10.7189/jogh.12.11009)
Supplement: Online Supplementary Document [file jogh-12-11009-s001.pdf]

Online Supplementary Document

Figure S1 Directed acyclic graph for the association between maternal active smoking in 3 months before pregnancy and risk of CHDs in offspring.

Figure S2 Directed acyclic graph for the association between maternal passive smoking in 3 months before pregnancy and risk of CHDs in offspring.

Figure S3 Directed acyclic graph for the association between maternal active smoking in early pregnancy and risk of CHDs in offspring.

Figure S4 Directed acyclic graph for the association between maternal passive smoking in early pregnancy and risk of CHDs in offspring.

Figure S5 The risks of CHDs in offspring of pregnant women with active smoking or passive smoking in 3 months before pregnancy after excluding pregnant women whose children had non-cardiac defects.

Figure S6 The risks of CHDs in offspring of pregnant women with active smoking or passive smoking in early pregnancy after excluding pregnant women whose children had non-cardiac defects.

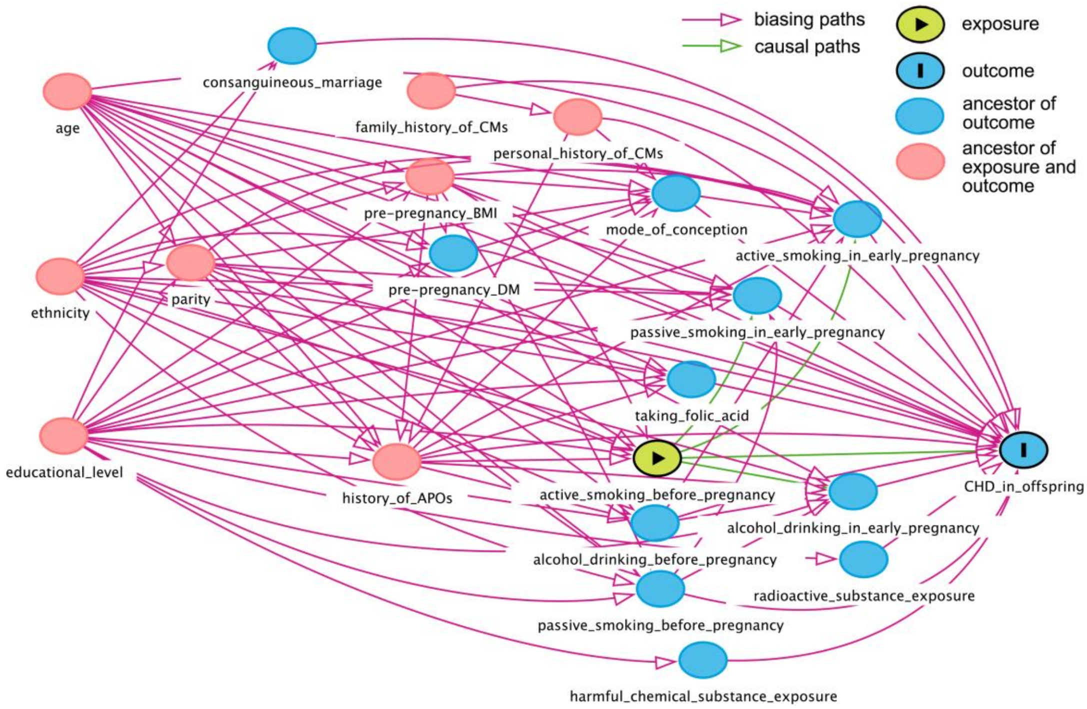

Figure S1 Directed acyclic graph for the association between maternal active smoking in 3 months before pregnancy and risk of CHDs in offspring.

Minimal sufficient adjustment sets for estimating the effect of maternal active smoking in 3 months before pregnancy on offspring CHDs: age, ethnicity, educational level, parity, history of adverse pregnancy outcomes, and pre-pregnancy BMI.

APOs, adverse pregnancy outcomes; BMI, body mass index; CHDs, congenital heart diseases; CMs, congenital malformations; DM, diabetes mellitus.

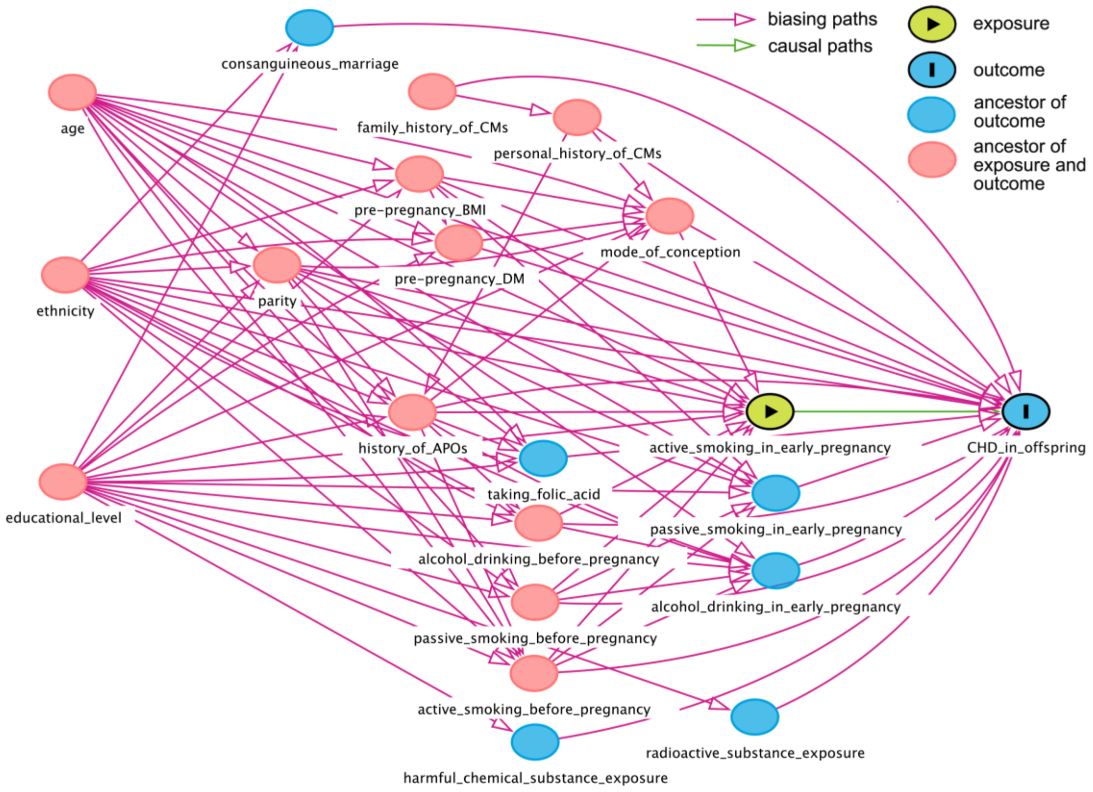

Figure S2 Directed acyclic graph for the association between maternal passive smoking in 3 months before pregnancy and risk of CHD in offspring.

Minimal sufficient adjustment sets for estimating the effect of maternal passive smoking in 3 months before pregnancy on offspring CHD: age, ethnicity, educational level, parity, and history of adverse pregnancy outcomes.

APOs, adverse pregnancy outcomes; BMI, body mass index; CHD, congenital heart disease; CMs, congenital malformations; DM, diabetes mellitus.

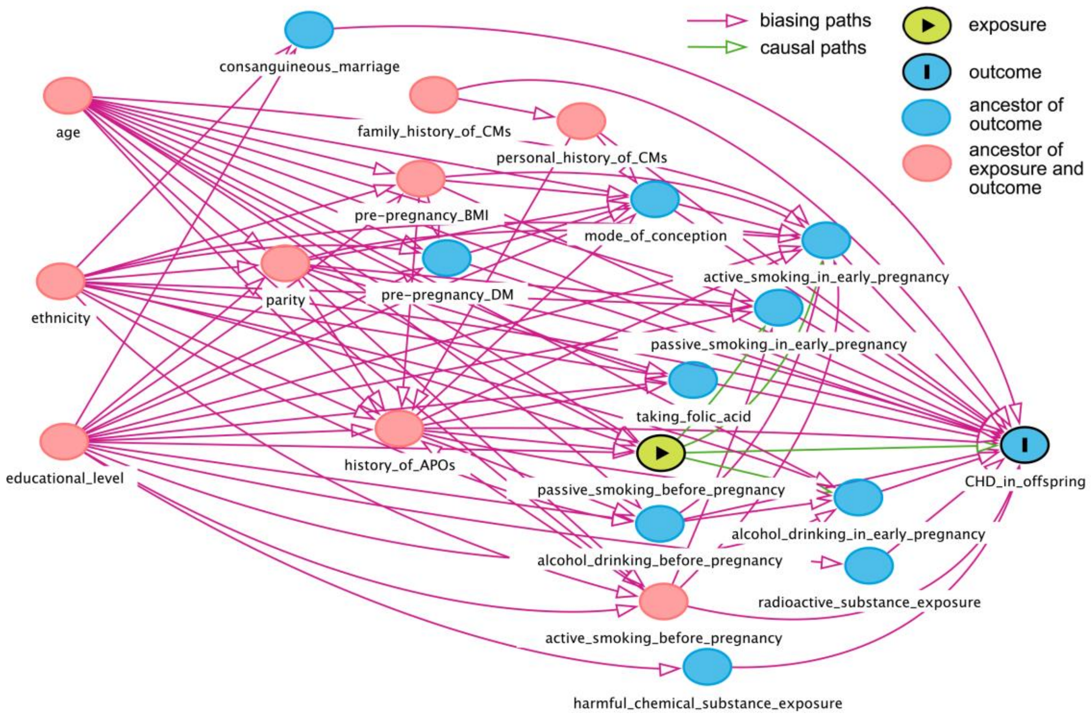

Figure S3 Directed acyclic graph for the association between maternal active smoking in early pregnancy and risk of CHD in offspring.

Minimal sufficient adjustment sets for estimating the effect of maternal active smoking in early pregnancy on offspring CHD: age, ethnicity, educational level, model of conception, parity, history of adverse pregnancy outcomes, pre-pregnancy BMI, active smoking in 3 months before pregnancy, passive smoking in 3 months before pregnancy, and alcohol consumption in 3 months before pregnancy.

APOs, adverse pregnancy outcomes; BMI, body mass index; CHD, congenital heart disease; CMs, congenital malformations; DM, diabetes mellitus.

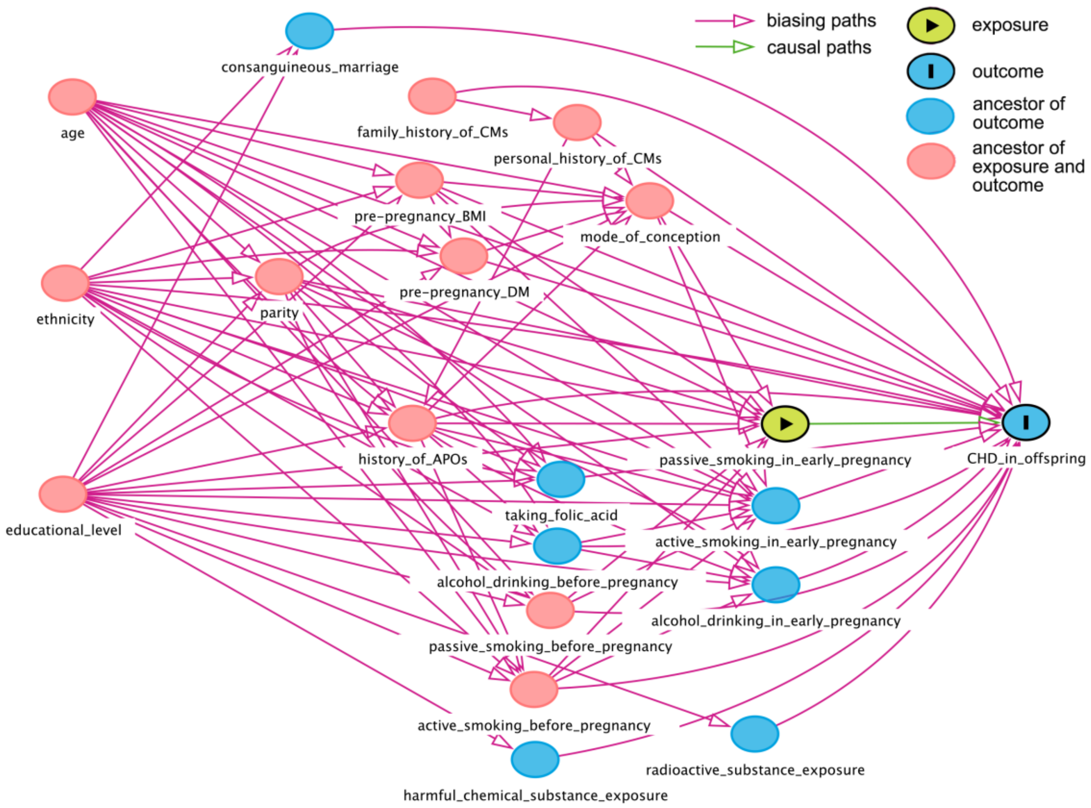

Figure S4 Directed acyclic graph for the association between maternal passive smoking in early pregnancy and risk of CHD in offspring.

Minimal sufficient adjustment sets for estimating the effect of maternal passive smoking in early pregnancy on offspring CHD: age, ethnicity, educational level, model of conception, parity, history of adverse pregnancy outcomes, pre-pregnancy BMI, active smoking in 3 months before pregnancy, and passive smoking in 3 months before pregnancy.

APOs, adverse pregnancy outcomes; BMI, body mass index; CHD, congenital heart disease; CMs, congenital malformations; DM, diabetes mellitus.

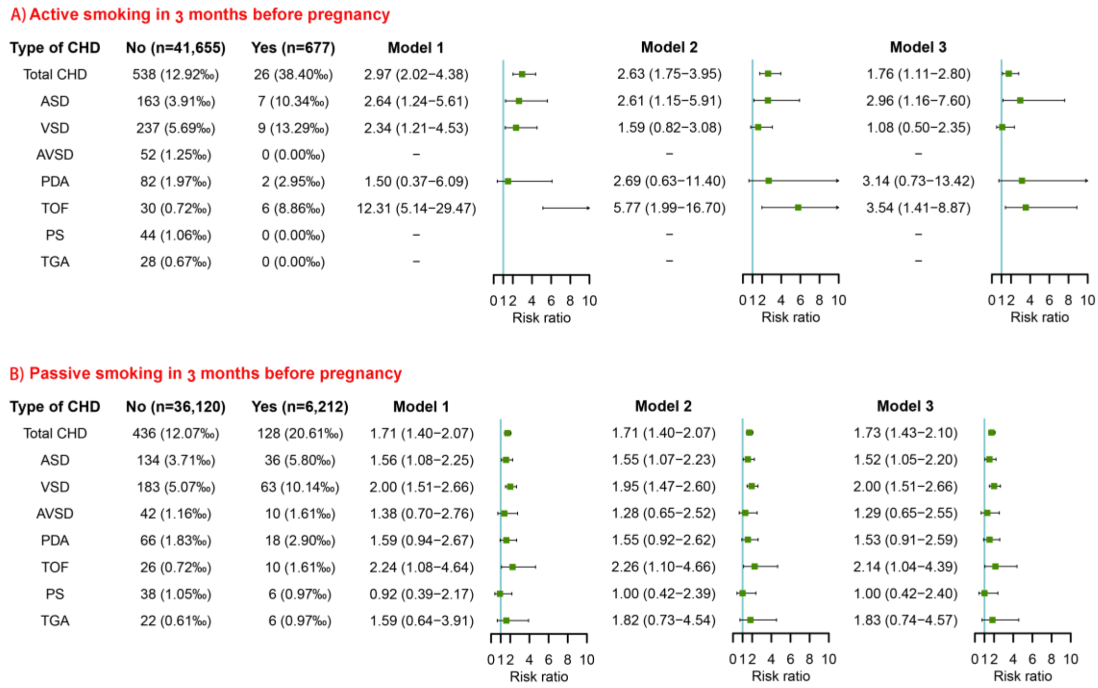

Figure S5 The risks of CHD in offspring of pregnant women with active smoking or passive smoking in 3 months before pregnancy after excluding pregnant women whose children had non-cardiac defects. **A)** Model 1 was a crude model without any variable adjusted; model 2 adjusted for age, ethnicity, educational level, parity, history of adverse pregnancy outcomes, and pre-pregnancy BMI; model 3 adjusted for age, ethnicity, educational level, parity, history of adverse pregnancy outcomes, pre-pregnancy BMI, passive smoking in 3 months before pregnancy, and alcohol drinking in 3 months before pregnancy. **B**) Model 1 was a crude model without any variable adjusted; model 2 adjusted for age, ethnicity, educational level, parity, and history of adverse pregnancy outcomes; model 3 adjusted for age, ethnicity, educational level, parity, history of adverse pregnancy outcomes, active smoking in 3 months before pregnancy, and alcohol drinking in 3 months before pregnancy.

CHDs, congenital heart defects; BMI, body mass index.

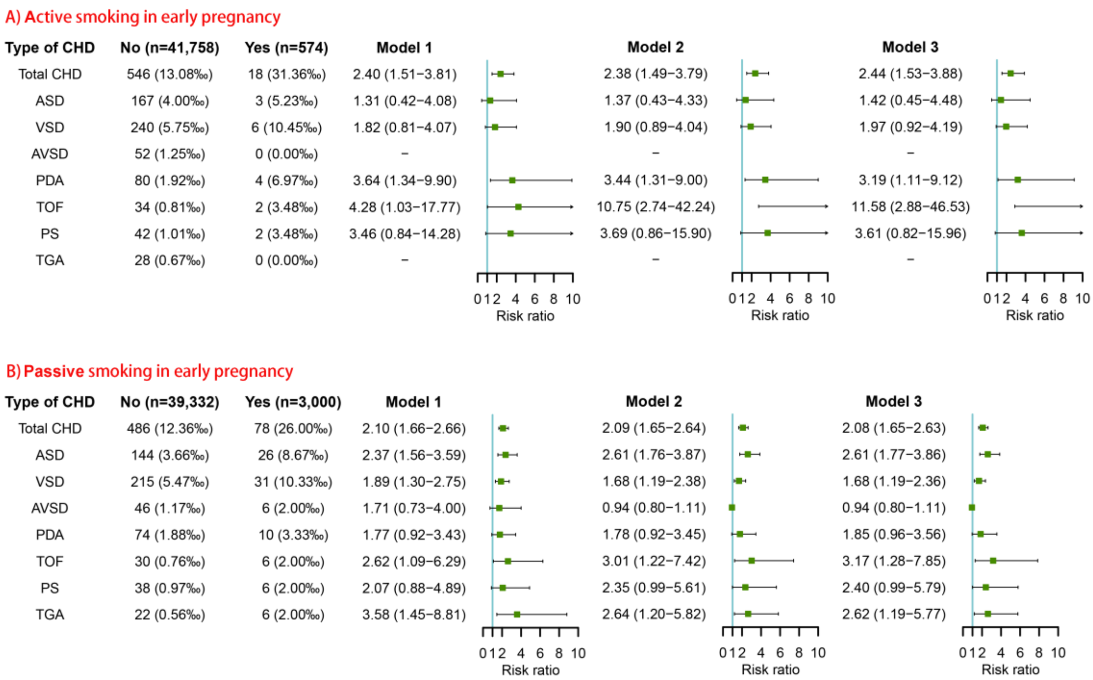

A) Active smoking in early pregnancy

Type of CHD	No (n=41,758)	Yes (n=574)	Model 1	Model 2	Model 3
Total CHD	546 (13.08‰)	18 (31.36‰)	2.40 (1.51–3.81)	2.38 (1.49–3.79)	2.44 (1.53–3.88)
ASD	167 (4.00‰)	3 (5.23‰)	1.31 (0.42–4.08)	1.37 (0.43–4.33)	1.42 (0.45–4.48)
VSD	240 (5.75‰)	6 (10.45‰)	1.82 (0.81–4.07)	1.90 (0.89–4.04)	1.97 (0.92–4.19)
AVSD	52 (1.25‰)	0 (0.00‰)	–	–	–
PDA	80 (1.92‰)	4 (6.97‰)	3.64 (1.34–9.90)	3.44 (1.31–9.00)	3.19 (1.11–9.12)
TOF	34 (0.81‰)	2 (3.48‰)	4.28 (1.03–17.77)	10.75 (2.74–42.24)	11.58 (2.88–46.53)
PS	42 (1.01‰)	2 (3.48‰)	3.46 (0.84–14.28)	3.69 (0.86–15.90)	3.61 (0.82–15.96)
TGA	28 (0.67‰)	0 (0.00‰)	–	–	–

B) Passive smoking in early pregnancy

Type of CHD	No (n=39,332)	Yes (n=3,000)	Model 1	Model 2	Model 3
Total CHD	486 (12.36‰)	78 (26.00‰)	2.10 (1.66–2.66)	2.09 (1.65–2.64)	2.08 (1.65–2.63)
ASD	144 (3.66‰)	26 (8.67‰)	2.37 (1.56–3.59)	2.61 (1.76–3.87)	2.61 (1.77–3.86)
VSD	215 (5.47‰)	31 (10.33‰)	1.89 (1.30–2.75)	1.68 (1.19–2.38)	1.68 (1.19–2.36)
AVSD	46 (1.17‰)	6 (2.00‰)	1.71 (0.73–4.00)	0.94 (0.80–1.11)	0.94 (0.80–1.11)
PDA	74 (1.88‰)	10 (3.33‰)	1.77 (0.92–3.43)	1.78 (0.92–3.45)	1.85 (0.96–3.56)
TOF	30 (0.76‰)	6 (2.00‰)	2.62 (1.09–6.29)	3.01 (1.22–7.42)	3.17 (1.28–7.85)
PS	38 (0.97‰)	6 (2.00‰)	2.07 (0.88–4.89)	2.35 (0.99–5.61)	2.40 (0.99–5.79)
TGA	22 (0.56‰)	6 (2.00‰)	3.58 (1.45–8.81)	2.64 (1.20–5.82)	2.62 (1.19–5.77)

Figure S6 The risks of CHD in offspring of pregnant women with active smoking or passive smoking in early pregnancy after excluding pregnant women whose children had non-cardiac defects. **A)** Model 1 was a crude model without any variable adjusted; model 2 adjusted for age, ethnicity, educational level, model of conception, parity, history of adverse pregnancy outcomes, pre-pregnancy BMI, active smoking in 3 months before pregnancy, passive smoking in 3 months before pregnancy, and alcohol consumption in 3 months before pregnancy; model 3 adjusted for age, ethnicity, educational level, model of conception, parity, history of adverse pregnancy outcomes, pre-pregnancy BMI, active smoking in 3 months before pregnancy, passive smoking in 3 months before pregnancy, alcohol consumption in 3 months before pregnancy, passive smoking in early pregnancy, and alcohol drinking in early pregnancy. **B**) Model 1 was a crude model without any variable adjusted; model 2 adjusted for age, ethnicity, educational level, model of conception, parity, history of adverse pregnancy outcomes, pre-pregnancy BMI, active smoking in 3 months before pregnancy, and passive smoking in 3 months before pregnancy; model 3 adjusted for age, ethnicity, educational level, model of conception, parity, history of adverse pregnancy

outcomes, pre-pregnancy BMI, active smoking in 3 months before pregnancy, passive smoking in 3 months before pregnancy, active smoking in early pregnancy, and alcohol drinking in early pregnancy.

CHDs, congenital heart defects; BMI, body mass index.
